# Insights into taxadiene synthase catalysis and promiscuity facilitated by mutability landscape and molecular dynamics

**DOI:** 10.1007/s00425-024-04363-9

**Published:** 2024-03-09

**Authors:** Siqi He, Ingy I. Abdallah, Ronald van Merkerk, Wim J. Quax

**Affiliations:** 1 Department of Chemical and Pharmaceutical Biology, Groningen Research Institute of Pharmacy, University of Groningen, Antonius Deusinglaan 1, 9713 AV Groningen, The Netherlands; 2 Department of Pharmacognosy, Faculty of Pharmacy, Alexandria University, Alexandria, Egypt

**Keywords:** AlphaFold model, Catalytic activity, Mutagenesis, Product profile, Terpenoid

## Abstract

**Main conclusion:**

Protein modeling, carbocation docking, and molecular dynamics along with structure-based mutability landscapes provided insight into taxadiene synthase catalysis (first step of the anticancer Taxol biosynthesis), protein structure–function correlations, and promiscuity.

**Abstract:**

Plant terpenes belong to one of the largest and most diverse classes of natural products. This diversity is driven by the terpene synthase enzyme family which comprises numerous different synthases, several of which are promiscuous. Taxadiene synthase (TXS) is a class I diterpene synthase that catalyzes the first step in the biosynthesis pathway of the diterpene Taxol, an anticancer natural product produced by the *Taxus* plant. Exploring the molecular basis of TXS catalysis and its promiscuous potential garnered interest as a necessary means for understanding enzyme evolution and engineering possibilities to improve Taxol biosynthesis. A catalytically active closed conformation TXS model was designed using the artificial intelligence system, AlphaFold, accompanied by docking and molecular dynamics simulations. In addition, a mutability landscape of TXS including 14 residues was created to probe for structure–function relations. The mutability landscape revealed no mutants with improved catalytic activity compared to wild-type TXS. However, mutations of residues V584, Q609, V610, and Y688 showed high degree of promiscuity producing cembranoid-type and/or verticillene-type major products instead of taxanes. Mechanistic insights into V610F, V584M, Q609A, and Y688C mutants compared to the wild type revealed the trigger(s) for product profile change. Several mutants spanning residues V584, Q609, Y688, Y762, Q770, and F834 increased production of taxa-4(20),11(12)-diene which is a more favorable substrate for Taxol production compared to taxa-4(5),11(12)-diene. Finally, molecular dynamics simulations of the TXS reaction cascade revealed residues involved in ionization, carbocation stabilization, and cyclization ushering deeper understanding of the enzyme catalysis mechanism.

**Graphical abstract:**

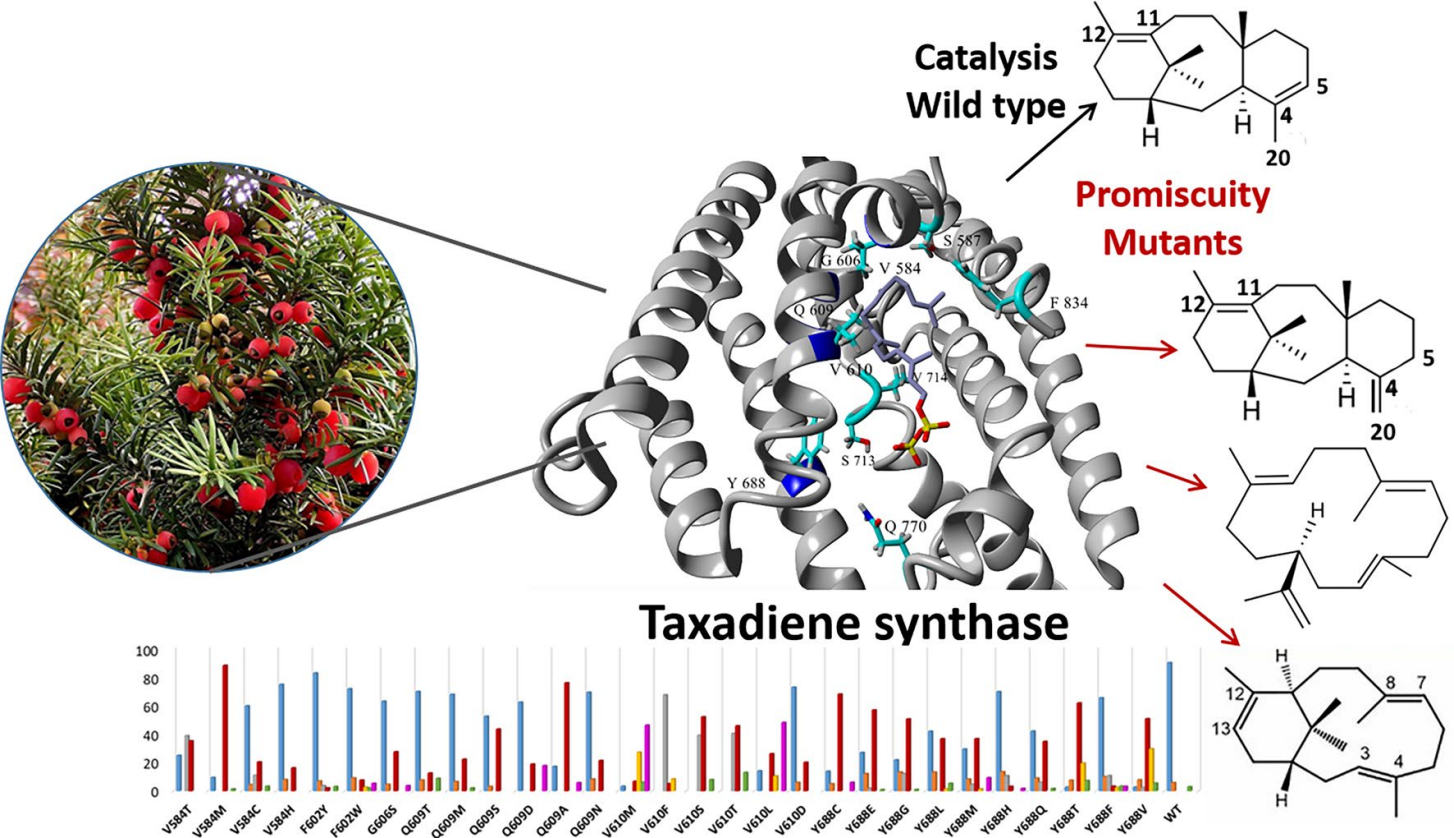

**Supplementary Information:**

The online version contains supplementary material available at 10.1007/s00425-024-04363-9.

## Introduction

Terpenes constitute the largest class among the myriad of small molecule natural products that are broadly spread through all domains of life. Plants yield considerably the greatest array of diverse terpenoid structures, in the scores of tens of thousands, with various roles. Terpene synthase enzymes are pivotal players affecting terpene diversity. Unraveling the metabolic enzymes that create terpene chemical diversity became a long standing research concern due to the biological and economic importance of terpenes. In addition, understanding the function and development of the terpene synthase family can give insight into plant evolution (Chen et al. 2011; Pichersky and Raguso 2018; Karunanithi and Zerbe 2019; Jia et al. 2022). Terpenes are characterized by their complex structures as they all originate from common isoprenyl building blocks. The reaction cascades by which terpenes are synthesized are based on elongation of acyclic poly-isoprenyl precursors accompanied with the hallmark carbocationic-driven cyclization and/or rearrangement which is the main cause of the complexity of their structures. The basic structures of all these different terpenes are determined by the involved terpene synthase enzymes. Terpene diversity is mainly attributed to the numerous diverse terpene synthases involved in their biosynthesis, in addition to the promiscuity of some terpene synthases producing multiple products (Bohlmann et al. 1998; Degenhardt et al. 2009; Gao et al. 2012; Christianson 2017). Hence, research into enzyme structure–function relationships of terpene synthases underlying their complex mechanism cascade garnered increased interest.

The enzyme taxadiene synthase (TXS), a class I diterpene synthase, is responsible for the catalysis of the first committed step toward the biosynthesis of the diterpene Taxol, a well-known anticancer plant natural product produced by the *Taxus* plant. Diterpene synthases comprise both class I and class II enzymes. The structure of diterpene synthases is composed of three domains designated αβγ. Reactions by class I diterpene synthases occur solely in the α domain, while those by class II enzymes take place at the interface of the β and γ domains. TXS, as a class I diterpene synthase, catalyzes the cyclization of geranylgeranyl pyrophosphate (GGPP), the universal diterpene precursor, to taxa-4(5),11(12)-diene (taxa-4(5)-diene) as a major product in the α domain of the enzyme. Full length TXS consists of 862 residues, including an N-terminal plastid targeting sequence of ≈ 80 residues which is usually deleted to express a pseudo mature form of TXS. Assembled into three α-helical domains, the active site of TXS is positioned in the C-terminal domain (S553-V862) comprising the metal-binding motifs D^613^DMAD^617^ and N^757^DTKT^761^YQAE^765^ that coordinate three Mg^+2^ ions, typical to a class I terpene synthase (Koksal et al. 2011; Gao et al. 2012; Pemberton et al. 2017).

**Fig. 1:**
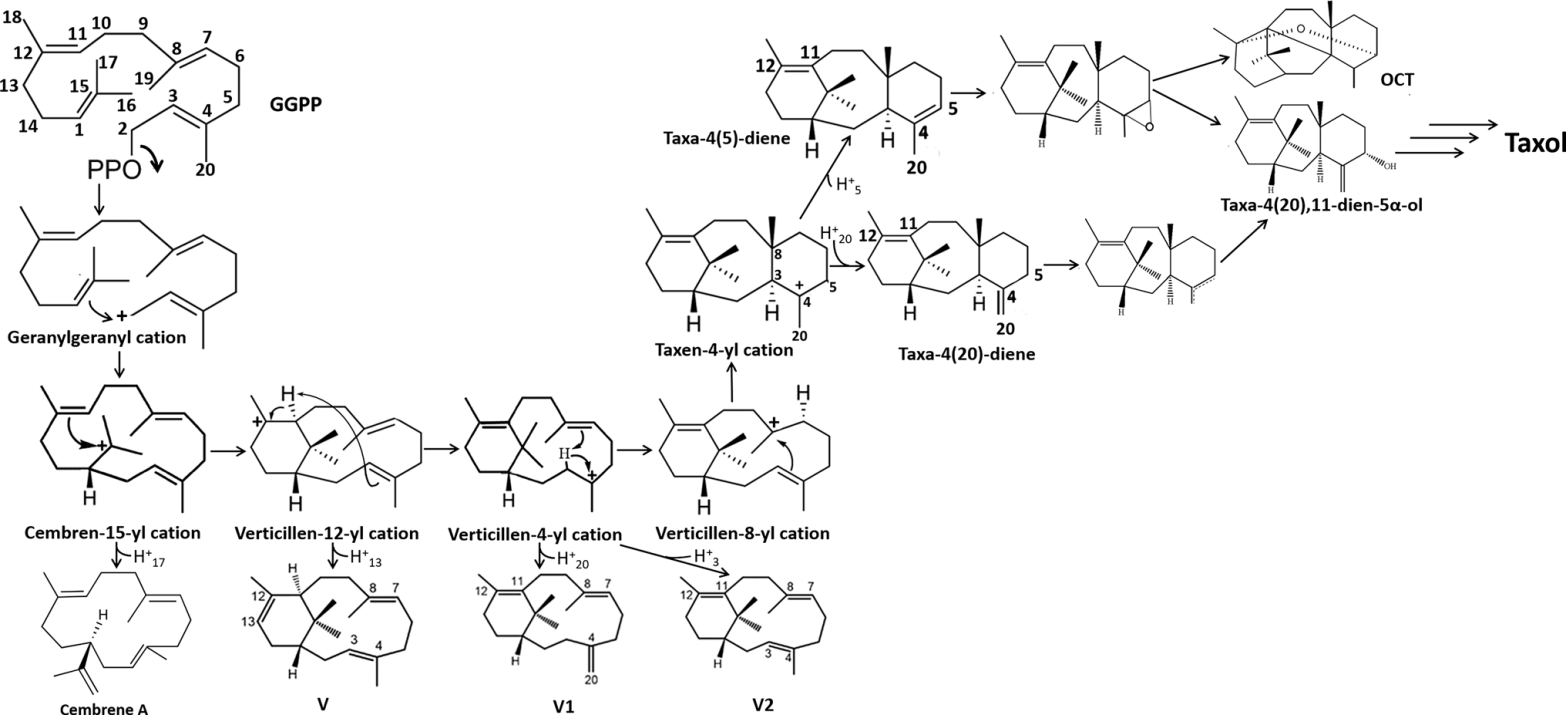
Proposed reaction cascade of taxadiene synthase catalysis toward Taxol biosynthesis. GGPP: geranylgeranyl pyrophosphate; V: verticillia-3(4),7(8),12(13)-triene; V1: verticillia-4(20),7(8),11(12)-triene; V2: verticillia-3(4),7(8),11(12)-triene; Taxa-4(5)-diene: taxa-4(5),11(12)-diene; Taxa-4(20)-diene: taxa-4(20),11(12)-diene

Being a class I terpene synthase, TXS reaction cascade (Fig. 1) starts with metal-triggered ionization of the substrate (GGPP) pyrophosphate (PPi) group. This leads to the creation of the highly reactive geranylgeranyl carbocation followed by multistep rearrangements, proton transfers, and cyclizations of the resulting carbocations leading to the production of the major product taxa-4(5)-diene with other minor products along the reaction cascade namely; taxa-4(20),11(12)-diene (taxa-4(20)-diene), cembrene A, verticillia-3(4),7(8),12(13)-triene (V), verticillia-4(20),7(8),11(12)-triene (V1), and verticillia-3(4),7(8),11(12)-triene (V2). This is the first committed step toward the production of Taxol. Following that, cytochrome p450s are responsible for oxygenation of the taxadiene skeleton at multiple locations, in addition to acylation via acyltransferases to synthesize Taxol. Cytochrome p450 taxadiene-5α-hydroxylase (CYP725A4) reacts differently toward the two isomers; taxa-4(5)-diene and taxa-4(20)-diene. Taxa-4(5)-diene is transformed to 5(12)-oxa-3(11)-cyclotaxane (OCT) as the major product with minor amount of taxa-4(20),11(12)-dien-5α-ol, the desired product for Taxol biosynthesis, while taxa-4(20)-diene is exclusively hydroxylated with a high degree of specificity to taxa-4(20),11(12)-dien-5α-ol. Based on literature, a proposed reaction cascade of TXS catalysis toward Taxol biosynthesis is shown in Fig. 1 (Lin et al. 1996; Jin et al. 2005; Koksal et al. 2011; Hong and Tantillo 2011; Schrepfer et al. 2016; Edgar et al. 2017; Ansbacher et al. 2018; Escorcia et al. 2018).

TXS enzyme controls and guides the reaction cascade through the unstable intermediate carbocations toward its product taxadiene while attempting to minimize side reactions. Understanding the molecular basis of TXS catalysis and its promiscuous potential is of great interest as an essential tool for unraveling enzyme evolution and possible engineering to further improve the biosynthesis process of Taxol. The aim of the current study is to gain a more comprehensive understanding of TXS catalysis and the active site residues involved in its reaction cascade. A mutability landscape of selected active site residues of TXS was created to explore their role in TXS mechanism and the impact of their mutation on activity and product profile. It was revealed that the promiscuity of TXS, as several terpene synthases, was amplified by some mutations. This research provides further insight into the mechanistic structure–function relationships of the enzyme and its promiscuous nature pinpointing essential active site residues for TXS reaction cascade, in addition to, residues that can alter the major product of TXS in favor of one of its minor side products. It also contributes to the efforts of engineering TXS as a means of overcoming bottle necks in the Taxol biosynthesis process.

## Materials and methods

### Creation of TXS model and selection of residues for mutation

AlphaFold2 Colab was employed to predict the structure of TXS from its amino acid sequence (Jumper et al. 2021; Varadi et al. 2022) followed by an extra relaxation step with AMBER to ensure accurate location of the side chains of the residues. AlphaFill was used to enrich the TXS AlphaFold model with magnesium ions and pyrophosphate group based on what have been experimentally detected in homologous protein structures (Hekkelman et al. 2023). Quantum mechanical/molecular mechanical (QM/MM) method was used to obtain more accurate atomic charges of ligands for docking calculations by considering the polarization effects of enzymes on ligands. Gaussian09 software was applied for restrained electrostatic potential (RESP) charge for quantum mechanics optimization of the partial charges of the different carbocation intermediates in preparation for their docking (Bayly et al. 1993; Frisch et al. 2009; Delgado-Arciniega et al. 2023). AutoDockVina was utilized for docking GGPP and the intermediate carbocations in the active site of the generated TXS model (Trott and Olson 2010; Eberhardt et al. 2021). Finally, 20 ns molecular dynamics (MD) simulations (Wijma et al. 2014) were performed using YASARA (version 23.5.19; YASARA Biosciences). The molecular dynamics simulation study was conducted with the YASARA dynamics software package (Land and Humble 2018) with the aid of the YAMBER3 force field. The enzyme–substrate complexes were initially cleaned and minimized, and placed within a rectangular simulation cell. The distances between the protein and the periodic boundaries of the cell were maintained at a minimum of 7.5 Å (Wijma et al. 2014). MD simulations were employing a leapfrog integration scheme and a Berendsen thermostat (Berendsen et al. 1984; Krieger et al. 2004) under pressure control. The physiological conditions of the simulation cells were set as 298 K, pH 7.4, and 0.9% NaCl. The temperature was gradually increased from 5 to 298 K during 30 ps, after which the simulation was allowed to equilibrate for 70 ps before the production phase simulation of 20,000 ps. Snapshots were obtained every 25 ps. The active site of the TXS model with docked GGPP was examined using YASARA to detect interactions and select candidate residues for mutation. In addition, MD simulations of the docked geranylgeranyl, cembren-15-yl and verticillen-12-yl cations gave insight into the first steps of the reaction cascade. Similarly, interesting TXS mutant models were created and examined.

### Creation of TXS mutant library

A truncated txs gene from the plant *Taxus baccata* (GeneBank/NCBI accession number AY424738) cloned in frame with the N-terminal histidine tag of pET15b vector was utilized as the template. Randomization of each residue was performed through the small-intelligent strategy (Tang et al. 2012; Abdallah et al. 2018) where a mixture of four pairs of complementary primers (Eurofins, Ebersberg, Germany) with the degenerate codons/codons NDT, VMA, ATG, and TGG was used at a ratio of 12:6:1:1, respectively. The library was generated using Quikchange method where PCR reactions (50 μl) with the primer mixture were executed using Phusion high fidelity DNA polymerase and Phusion GC buffer followed by digestion with DpnI and transformation into competent *E. coli* XL1-blue. The generated colonies per residue were sequenced by GATC Biotech. Following sequencing, each chosen colony was individually grown in Luria − Bertani (LB) broth supplemented with 100 μg/ml ampicillin. Through the NucleoSpin 96 Plasmid Core Kit (BIOKÉ, Leiden, The Netherlands), each plasmid containing a distinct mutant TXS gene was isolated from its corresponding culture. After that, the isolated plasmids were separately transformed into chemically competent *E. coli* BL21 (DE3). The mutant library consisting of *E. coli* BL21 transformants, each harboring a pET15b vector with a unique TXS gene, was stored at -80 ºC till later use.

### Expression, purification, and quantitation of the TXS mutant library

The *E. coli* BL21 (DE3) strains retaining TXS mutant genes were inoculated into 1 ml LB medium supplemented with 100 μg/ml ampicillin and incubated at 37 °C overnight. The overnight cultures were diluted, in 15 ml auto-induction medium [phosphate buffer (pH 7.2), 0.5% yeast extract, 2% tryptone, 1% NaCl, 0.6% glycerol, 0.05% glucose and 0.2% lactose] containing 100 μg/ml ampicillin, to OD_600_ of 0.05. The cultures were incubated at 37 ℃, 250 rpm till reaching OD_600_ of 0.7. Then they were grown overnight at 20 ℃, 190 rpm. The following day, the cultures were centrifuged for 10 min at 2100 g, 4 ℃ to collect the cell pellets. Then the cell pellets were subjected to three cycles of freezing and thawing followed by resuspension in 1.5 ml lysis buffer [50 mM Tris–HCl, pH 7.5–8.0, 100 mM NaCl, 10 mM β-mercaptoethanol, cOmplete™ EDTA-free protease inhibitor cocktail tablet (one tablet per 50 ml), 1 mg/ml lysozyme and 10 µg/ml DNase] and 30 min. incubation at 20 ℃, 250 rpm for cell lysis. Centrifugation for 10 min at 2100 g was performed to collect the soluble protein fractions. Finally, protein purification was executed through using His MultiTrap™ Fast Flow GE Healthcare 96-well plates with binding and wash buffer (20 mM Tris–HCl, 10 mM MgCl_2_, 150 mM NaCl, 1 mM DTT and 20 mM imidazole, pH 7.4–8), and addition of 10% glycerol and 250 mM imidazole for the elution buffer. SDS gels were used to visualize the purified protein library. After that, the concentrations of the mutants were determined using Thermo Scientific NanoDrop 1000 spectrophotometer and calculated based on molecular weight and extinction coefficient.

### Bioluminescent PPiLight™ inorganic pyrophosphate assay for TXS catalytic activity

The quantity of PPi produced from the conversion of the GGPP substrate to taxadiene by TXS is the measure used in this assay, where AMP is converted to ATP (by the produced PPi) which then reacts with luciferase enzyme releasing light (Eriksson et al. 2001). High-throughput screening of the library was facilitated using Perkin Elmer JANUS 8-tip Varispan Automated Liquid Handling Workstation. In opaque white wall flat bottom 96-well microtiter plates (Greiner LUMITRAC™ 600 microplates), 60 μl reactions were prepared by mixing 0.15 μM TXS protein and 37 μM GGPP in 10 mM Tris–HCl buffer (pH 7.4), 10 mM MgCl_2_, 2 mM DTT with 20 μl Lonza PPiLight™ converting and detection reagent mixture. The plates were instantaneously incubated in a 30 ºC preheated chamber of FLUOstar Omega Microplate Reader with moderate shaking. The luminescence signal was measured continually for 2 h and expressed as Relative Light Units (RLUs). RLUs/s was reported as the measure of the catalytic rate of reaction of each mutant and compared to that of the wild type.

### GC–MS assay for evaluation of the TXS mutant library product profile

The product profiles of the TXS mutants were determined through an in vitro GC–MS assay. Purified enzymes (1 µM) in 10 mM Tris–HCl buffer (pH 7.4), containing 10 mM MgCl_2_, 2 mM DTT, were assayed in 0.5 ml reactions with 20 μM GGPP substrate. The reactions were overlaid with 200 µl hexane containing tetradecane internal standard and incubated for 1.5 h at 30 ºC. Then the reactions were stopped by addition of 0.5 ml stop buffer (0.2 M KOH and 0.1 M EDTA). A Shimadzu GCMS-QP2010SE system equipped with a GC-2010 Plus high-performance gas chromatograph was used for the assay. Two microliters of each n-hexane extract was injected splitless on a HP-5MS (5%-phenyl)-methylpolysiloxane column (Agilent J&W 0.25 mm inner diameter, 0.25 µm thickness, 30 m length) using helium carrier gas and analyzed in total ion scan. The injector temperature was set to 250 °C; the oven initial temperature was adjusted to 50 °C with an increase of 5 °C/min till 180 °C and then an increment of 10 °C/min till 300 °C. The solvent cut-off was 5 min. The product profile of all reactions was compared to that of wild-type TXS. The NIST (National Institute of Standards and Technology, Gaithersburg, MD, USA) and other libraries were used for identification of different products by comparing their mass spectra to those of the libraries. The peak areas were estimated using the integration tools in GCMSsolution 1.20 software (Shimadzu, Den Bosch, The Netherlands) and corrected by means of the peak corresponding to the internal standard tetradecane.

## Results

### Closed conformation TXS model provides more dynamic insight compared to open conformation crystal structure

**Fig. 2:**
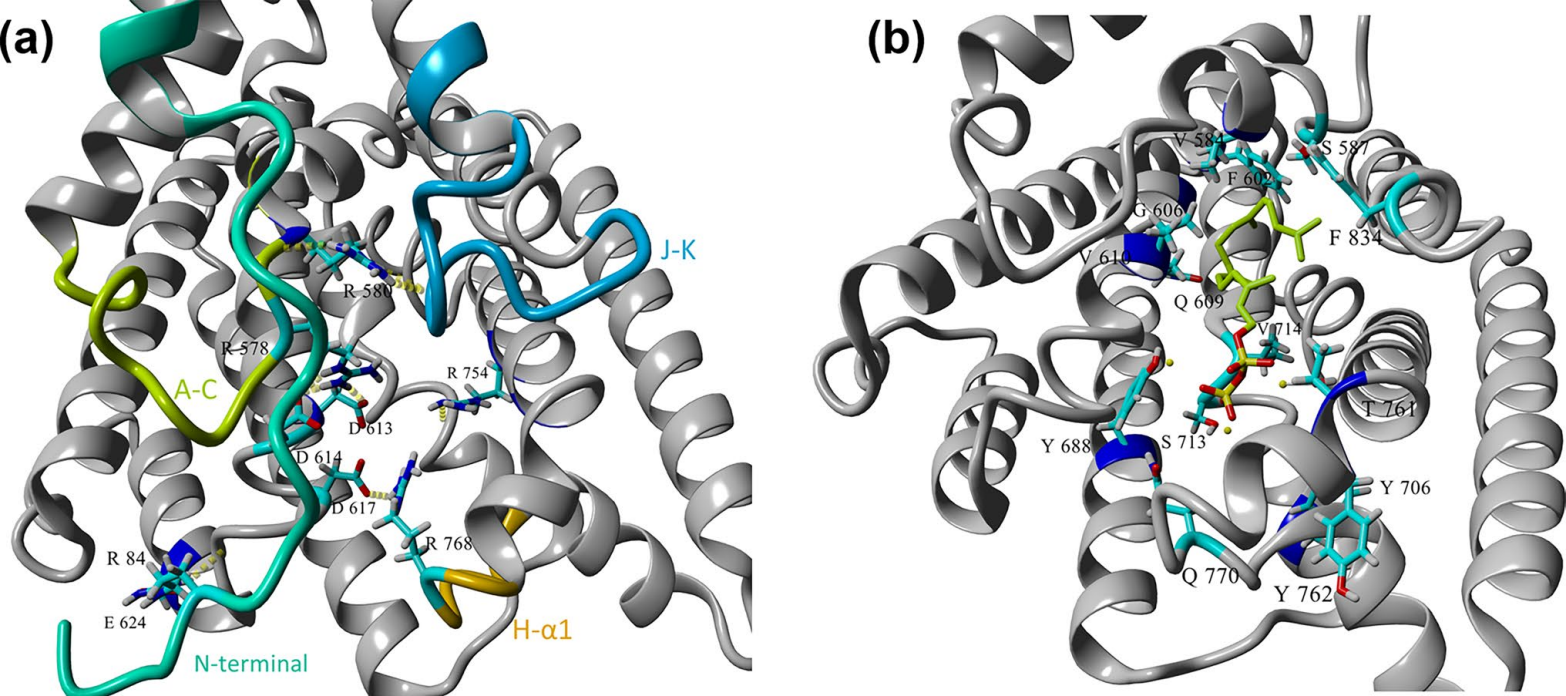
**a** TXS model showing N-terminal residues (80–110), the J-K loop (K836-D850), the A-C loop (S569-V581), and H-α1 loop (R768-S773) capping the active site. **b** The docked GGPP substrate and the active site residues selected for mutation

The currently published X-ray crystal structure of TXS (Koksal et al. 2011) is an open catalytically inactive form of the enzyme owing to the absence of the N-terminal residues, (80–110), that would both close the active site and keep a nearby loop in a place that caps the entrance of the active site so as to prevent the premature quenching of the reactive carbocations by the bulk solvent. Hence, the artificial intelligence system, AlphaFold, was employed to predict a closed catalytically active structure of TXS where the active site in the generated TXS model is capped by the N-terminal residues (80–110) and three loops; the J-K loop (K836-D850), the A-C loop (S569-V581) and H-α1 loop (R768-S773) (Fig. 2a) (Schrepfer et al. 2016; Freud et al. 2017; Escorcia et al. 2018). Our model is similar to the previously published Freud model (Freud et al. 2017) in the position and role of R578 and R580, that are known to be highly conserved among terpene synthases of class I, where R578 directly interacts with the pyrophosphate moiety, while R580 caps the active site by interacting with neighboring loops. Notably, this is different from the Schrepfer model (Schrepfer et al. 2016) where R580 is the residue interacting with the pyrophosphate moiety. The generated closed conformation TXS model was used for docking of the substrate and intermediate carbocations. Docking carbocations into the active site of terpene synthases is challenging (O’Brien et al. 2018; Raz et al. 2020). Docking produced poses with various orientations relative to the active site. These poses were clustered, and then accurate substrate folding and correct stereochemistry of the carbocations were considered. Molecular dynamics (MD) simulations were performed to reflect the natural TXS reaction cascade as closely as possible.

### Selection of residues for site-directed mutagenesis

The active site residues in the created TXS model with docked GGPP substrate were examined to choose candidate residues for mutation. Important residues can be located at any position in the enzyme structure; however, they are usually found inside or near the active site. Residues within 10 Å in the vicinity of the active site were initially considered, numbering around 84 residues. However, that radius included residues further from the substrate so these were eliminated bringing the number down to 58 residues within 7 Å radius. This number was narrowed down after considering predicted ligand–residue interactions, reports of important TXS residues in literature and sequence alignment with several terpene synthases to reveal conserved residues. Several conserved residues belonging to the two metal ion binding motifs were excluded. Also, residues that are mostly conserved among terpene synthases or were previously proven to be essential for activity (i.e., their mutation causes loss of activity) were excluded, such as R578, R580, W753, Y835, and Y841. Finally, the remaining residues were evaluated for their prospects based on TXS literature and the importance of the corresponding residues in other terpene synthases. Also, FireProt and Hotspot Wizard 3.0, in silico tools for automated and reliable design of thermostable mutant proteins depending on information about sequence, structure, and evolution, were used for computational predictions. Finally, 14 residues were selected for mutation (Fig. S1), namely V584, S587, F602, G606, Q609, V610, Y688, Y706, S713, V714, T761, Y762, Q770, and F834 (Fig. 2b). These choices were based on educated assumptions; nevertheless, other residues influencing enzyme activity maybe discovered in the future and some of the weeded out residues may later prove to be worthy of further research. Focusing the sequence alignments on the selected residues (Fig. S2) revealed that F602, Y688, Y706, Y762, and F834 are mainly conserved as aromatic, basic or strongly similar residue with few exceptions. S713, V714, T761, and Q770 tend to be conserved as identical or strongly similar residues with some exceptions. Unlike TXS, in the majority of the aligned sequences, V584 is mainly conserved as cysteine while S587 is conserved as tryptophan. G606, Q609, V610 are not conserved.

### Creation of TXS mutant library

A variety of TXS genes encoding different variants of the selected residues were created to express the mutability landscape of TXS. The “small-intelligent” strategy was utilized instead of the typical NNN or NNS(K) degenerate codons for site-directed mutagenesis. The “small-intelligent” strategy uses a set of four codons; NDT, VMA, ATG, and TGG allowing the exclusion of *E. coli* rare codons and preventing stop codons generation and amino acid biases. This method increases the quality of the mutant library produced (Tang et al. 2012; Abdallah et al. 2018). A mutant library of 266 variants was generated. The library was expressed in *E. coli* and purified based on the N-terminal his-tag of the proteins. The purified proteins were visualized on SDS gel to confirm expression and purity (Fig. S3).

### Mutability landscape of TXS for catalytic activity

**Fig. 3:**
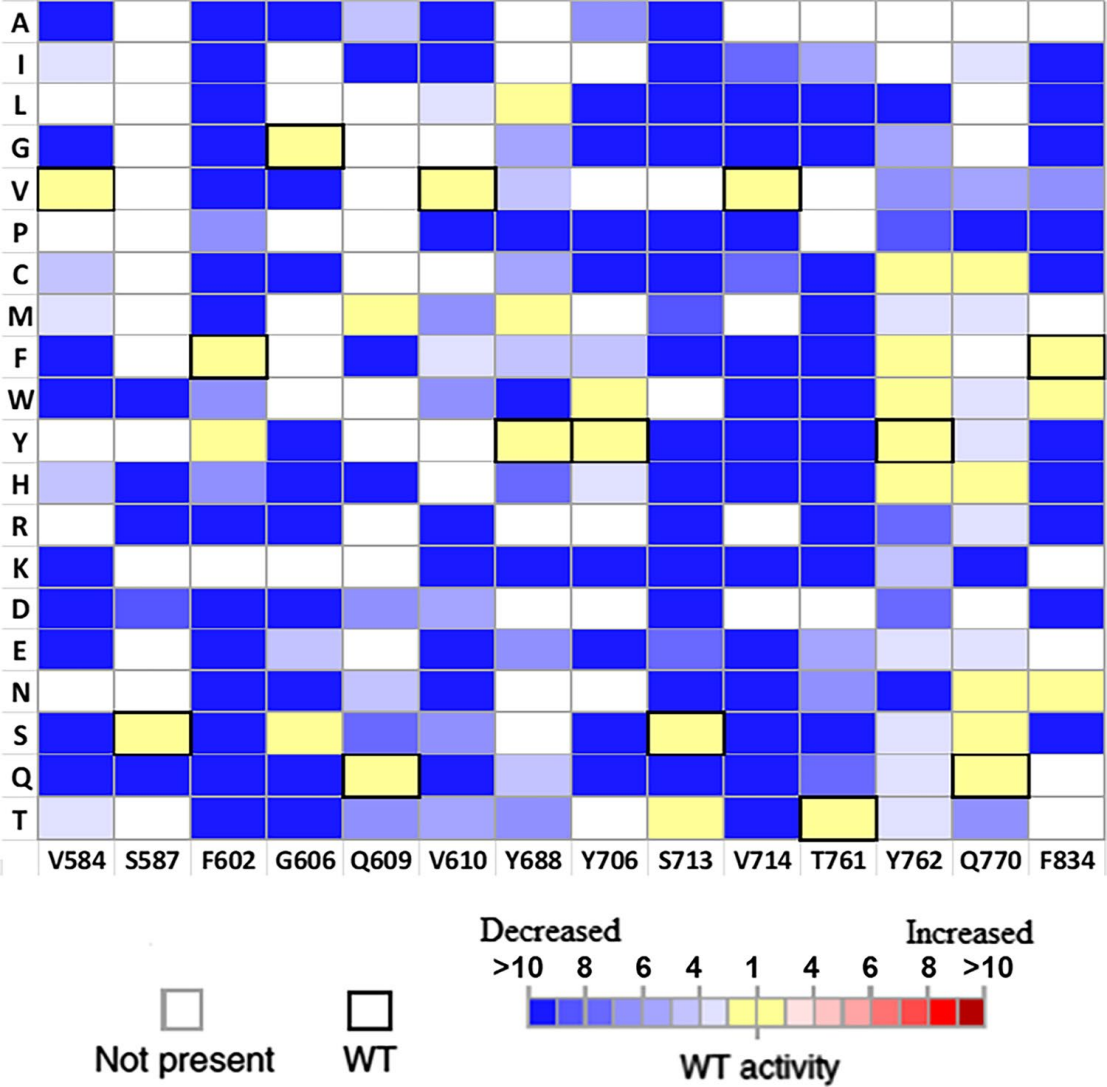
Mutability landscape of TXS for catalytic activity using bioluminescent assay. The vertical axis depicts the 20 possible amino acid residues. Bold squares highlight the wild-type amino acid residue at each position and variants that are absent from the library are represented by white squares. The color characterizes the catalytic rate of reaction (V) where blue range squares point to decreased catalytic activity while red range squares point to increased catalytic activity compared to the wild type and yellow squares represent activity similar to the wild type. The demonstrated data are an average of two isolated experiments (*n* = 2)

TXS transforms the substrate GGPP to taxa-4(5)-diene and some minor side products, releasing inorganic PPi during the process. A mutability landscape for the catalytic activity of TXS mutant library was created based on the fact that the amount of products created by TXS is equal to the amount of pyrophosphate released. Throughout the in vitro TXS reaction with GGPP, the quantity of pyrophosphate released was continuously measured by a rapid bioluminescent assay and represented as Relative Light Units (RLUs). The quantity of PPi released throughout the reaction is directly proportional to the quantity of light produced (Eriksson et al. 2001). Utilizing similar concentration of protein and GGPP, a curve of RLUs versus time was generated for all mutants in conjunction with wild-type TXS and the slope of the linear part was calculated to express the catalytic rate of reaction as RLUs/s (Abdallah et al. 2018). Based on the calculated slopes, a mutability landscape of the catalytic activity of the wild type compared to the mutants was created (Fig. 3). Mutants in the blue color range in the landscape showed decrease in enzyme activity relative to the wild type till complete loss of activity while mutants indicated with yellow color has activity comparable to the wild type and mutants showing shades of red color would indicate increase of activity relative to the wild type.

The mutability landscape discovered no mutants with improved activity compared to wild-type TXS. It was also revealed that most mutants of residues S587, F602, G606, Y706, S713, V714, T761, and F834 showed almost no enzymatic activity with some exceptions. Residue F602 retained comparable activity to wild-type TXS when mutated to tyrosine, tryptophan or histidine. Similar occurrence was observed when mutating residue Y706 to tryptophan, phenylalanine or histidine. Mutant of S713 showed similar activity when changed to threonine. Residue F834 retained activity similar to wild type when replaced by tryptophan or arginine. Numerous mutants of residues V584, Q609, V610, Y688, Y762 and Q770 showed activity comparable to or slightly lower than wild-type TXS with some of their mutants losing activity. It should be stressed that the landscape reflects only the pyrophosphate release. Further investigation of the enzyme activity in relation to the product profile of the mutants will be discussed.

### Assessment of the product profile of the TXS library using GC–MS

As a follow-up to the TXS library screening for catalytic activity, the GC–MS product profile of the mutants was assessed in comparison to that of wild-type TXS. The GC–MS chromatogram of wild-type TXS showed taxa-4(5)-diene as a major product with a ratio of approximately 90% relative to other minor products; taxa-4(20)-diene (≈ 6%), cembranoid-type and verticillene-type compounds (≈ 4%) (Fig. S4). The GC chromatograms of inactive mutants showed no products at all which corresponds with their catalytic inactivity revealed by the bioluminescent assay. GC–MS chromatograms were evaluated for mutants with increased production of taxa-4(20)-diene relative to the wild type which is considered a favorable isomer toward higher specificity for the final production of Taxol (Fig. 1) (Edgar et al. 2017). Also, product profiles were assessed for mutants showing higher production of cembranoid-type and verticillene-type compounds compared to the wild type where it was revealed that for some mutants, taxa-4(5)-diene was no longer the major product in favor of one of these compounds. The product profiles of the TXS library varied where several mutants resembled the wild-type profile with equivalent or reduced activity. Moreover, a number of mutants showed diverse product profiles clustered around a mixture of taxa-4(20)-diene, cembrene A, V, V1, V2 and an unknown product with mass spectrum and retention time closely related to V2 so identified as an isomer of V2 (Fig. S5–S11).

### Mutants with increased production of taxa-4(20)-diene

**Fig. 4:**
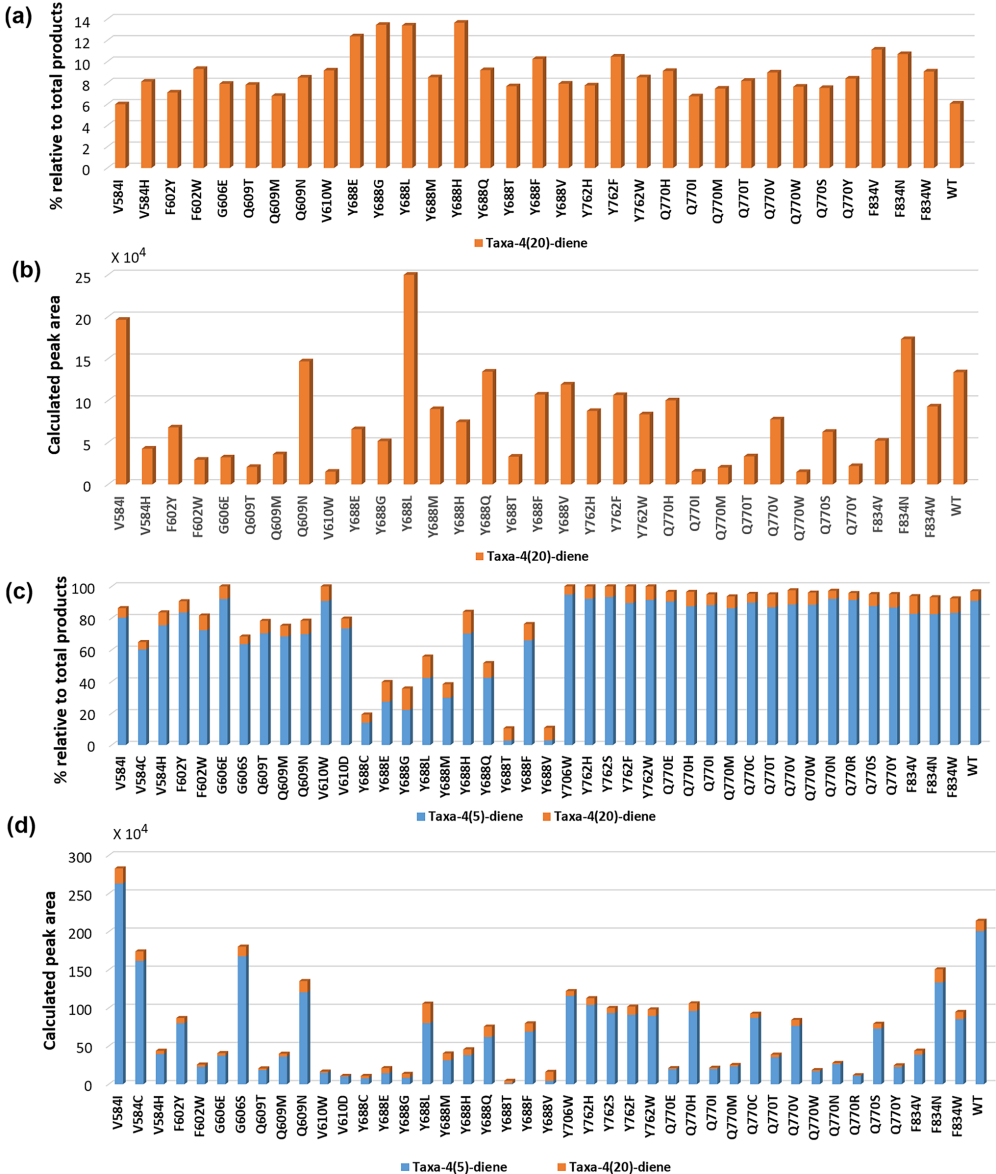
**a** Production of taxa-4(20)-diene by selected mutants depicted as % of taxa-4(20)-diene relative to the total % of products of the mutant. **b** Absolute amount of produced taxa-4(20)-diene represented by its calculated peak area. **c** Total production of taxa-4(5)-diene and taxa-4(20)-diene by selected mutants depicted as % of taxa-4(5)-diene and taxa-4(20)-diene relative to the total % of products of the mutant. **d** Absolute amount of produced taxa-4(5)-diene and taxa-4(20)-diene represented by their calculated peak areas. Note that the total % of products (100%) consists of the sum of % of taxa-4(5)-diene, taxa-4(20)-diene, cembranoid-type and verticillene-type compounds

The library was screened for mutants with increased production of taxa-4(20)-diene compared to wild-type TXS. Based on GC–MS chromatogram, wild-type TXS produced approximately 90% taxa-4(5)-diene and 6% taxa-4(20)-diene. Mutants showing taxa-4(20)-diene of 6% and higher (Fig. 4a) were evaluated. Mutants V584I, V584H, F602Y, F602W, G606E, Q609T, Q609M, Q609N, V610W, Y688M, Y688Q, Y688T, Y688V, Y762H, Y762W, Q770H, Q770I, Q77OM, Q770T, Q770V, Q770W, Q770S, Q770Y, and F834W produced 6–10% taxa-4(20)-diene relative to their total products. Mutants Y688E, Y688G, Y688L, Y688H, Y688F, Y762F, F834V, and F834N displayed higher taxa-4(20)-diene production up to 14% where Y688E, Y688G, Y688L, and Y688H produced 12.4%, 13.5%, 13.4%, and 13.7%, respectively. In addition, the absolute amount produced of taxa-4(20)-diene (Fig. 4b) was taken into consideration, represented by calculated peak area, and not only its percentage relative to the total products of each mutant. Although several mutants showed higher % of taxa-4(20)-diene, the absolute amount produced based on peak area was lower than that of wild-type TXS. This is probably due to their lower enzymatic activity compared to the wild type. To take advantage of the increased percentage of taxa-4(20)-diene by these mutants, additional mutations might have to be performed to improve enzymatic activity and boost the absolute production of taxa-4(20)-diene. Mutants V584I, Q609N, Y688L, and F834N exhibited higher absolute production of taxa-4(20)-diene where V584I and Y688L produced ≈1.5 and 2 times higher amounts compared to the wild type, respectively. Although, Y688L depicted high production of taxa-4(20)-diene compared to wild type, it showed much lower total production of both taxa-4(5)-diene and taxa-4(20)-diene (55% versus 96% of the wild type and almost half the absolute amount produced) (Fig. 4c and 4d). Other Y688 mutants displayed a similar pattern of low total production of taxa-4(5)-diene and taxa-4(20)-diene (Fig. 4) which can be attributed to increased production of other minor cembranoid-type and verticillene-type compounds. This pattern can have further impact on final production levels of Taxol.

### Mutants with increased production of cembrene A

**Fig. 5:**
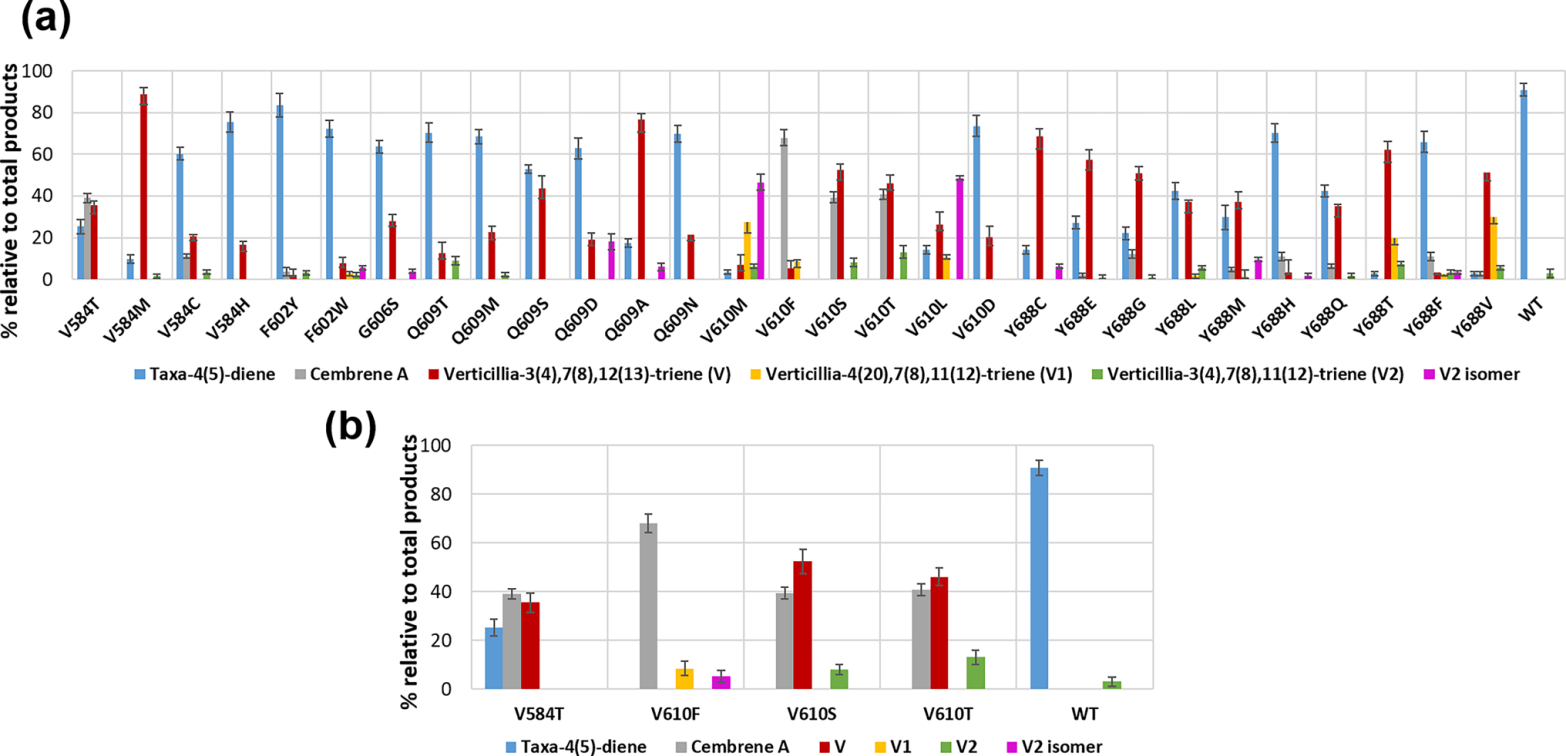
**a** Production of cembrene A and verticillene-type compounds by selected mutants demonstrated as % relative to the total % of products of the mutant. **b** Spotlight on mutants with high cembrene A production. Note that the total % of products (100%) consists of the sum of % of taxa-4(5)-diene, taxa-4(20)-diene, cembranoid-type and verticillene-type compounds. The demonstrated data are an average of three separate experiments (*n* = 3)

In the TXS library, some mutants displayed significantly increased production of the minor product cembrene A (Fig. 5) which was an undetectable minor product in the GC chromatogram of wild-type TXS. V584T showed decreased production of taxa-4(5)-diene (25%) with increased production of cembrene A (39%) and V (36%). V610F, V610S, and V610T stopped producing taxa-4(5)-diene completely where cembrene A became the major product of V610F (68%) along with minor production of V and V1 (Fig. S12). Similarly, V610S and V610T showed cembrene A and V as the major products along with minor production of V2.

### Mutants with increased production of verticillene-type compounds

The product profile of the TXS library was further assessed for mutants showing increased production of verticillene-type compounds (Fig. 5). Several mutants showed higher percentage of produced V, V1, and V2 compared to the wild type. Also, the GC chromatogram of some mutants showed a peak, at ≈ 20.85 min, of an unidentified product (Fig. S13). Since the mass spectrum (Fig. S11) correlated to that peak and its retention time are closely related to V2, it is suggested that the unidentified product is an isomer of V2. This increase in production of verticillene-type compounds is accompanied with decreased production of the major product taxa-4(5)-diene. V became the major product of mutants V584M (Fig. S14), Q609A (Fig. S15), V610S, V610T, Y688C (Fig. S16), Y688E, Y688G, Y688M, Y688T and Y688V with significantly reduced to almost no production of taxa-4(5)-diene. The newly identified V2 isomer was presented as the major product of mutants V610M and V610L. It is noteworthy that the residues from the TXS library that displayed the highest degree of promiscuity when mutated are V584, Q609, V610 and Y688. This can indicate that they play a crucial role in the catalytic mechanism of TXS.

## Discussion

**Fig. 6:**
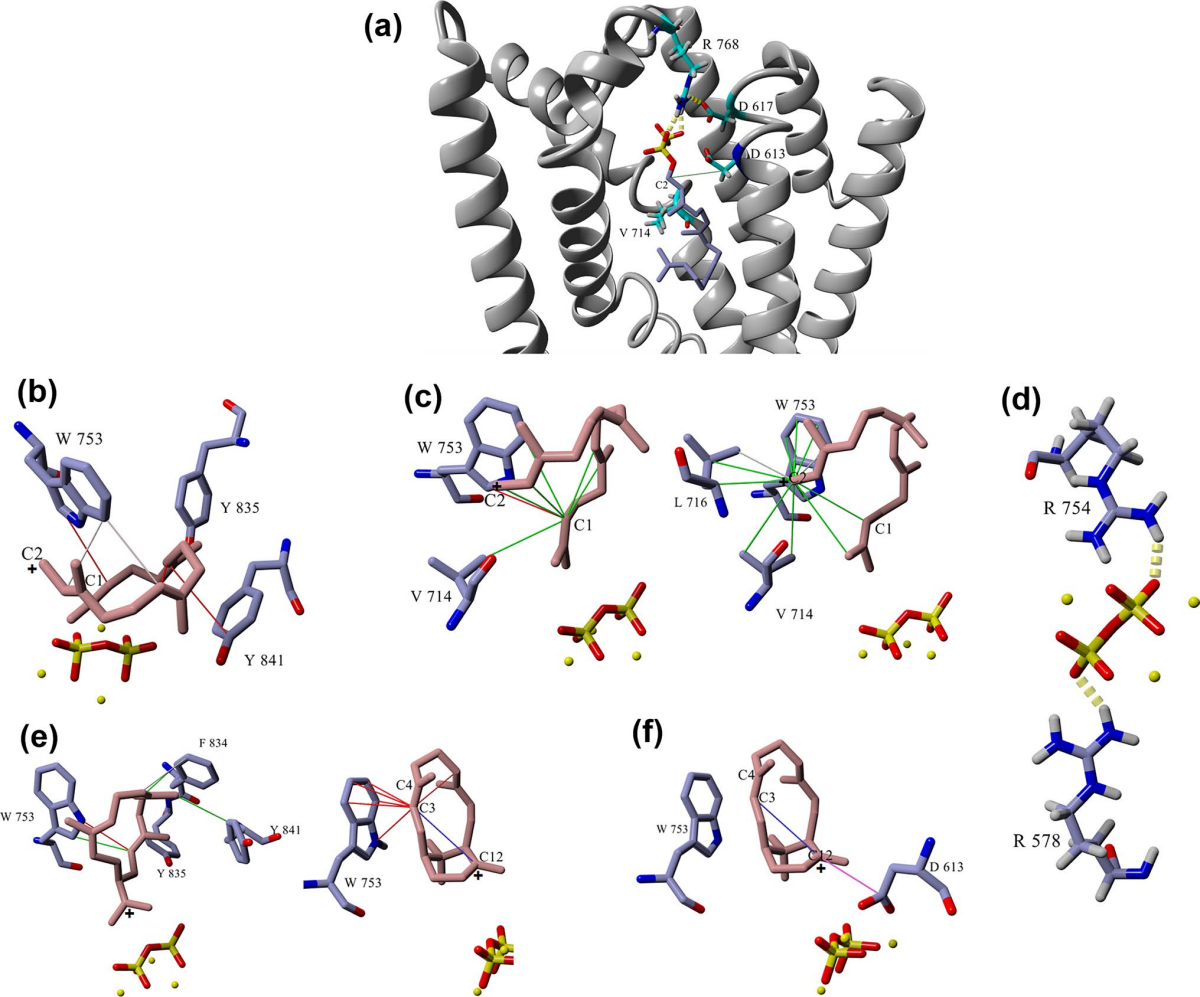
Close-up view of wild-type TXS active site with different docked ligands. **a** GGPP substrate surrounded by the effector triad of the PPi sensor R768, the linker D617, and the effector V714-O. **b** π–π interactions of W753, Y835, and Y841 with the geranylgeranyl cation. **c** Hydrophobic interactions of V714, L716, and W753 with C1 and C2 of the geranylgeranyl cation. **d** Suggested R578-PPi-R754 motif. **e** π–π interactions of W753, F834, Y835, and Y841 with the cembren-15-yl cation and π–π interactions of W753 with the verticillen-12-yl cation. **f** Verticillen-12-yl cation–π interaction between C3–C4 double bond and the positively charged C12 which in turn show ionic interaction with D613

Terpene synthases control complex multistep carbocation cascades with stereo- and regio-chemical specificity producing numerous structurally diverse terpenes. In-depth structure–function studies utilizing protein modeling, carbocation docking and molecular dynamics along with structure-based mutability landscapes provide more precise functional prediction of terpene synthase catalysis and promiscuity (Karunanithi and Zerbe 2019). In the present study, we utilized protein modeling, carbocation docking, molecular dynamics simulations and mutability landscape to gain deeper insight into the catalysis, protein structure–function associations and promiscuity of the interesting diterpene synthase, taxadiene synthase. TXS reaction cascade starts with trinuclear Mg^+2^ triggered ionization of GGPP which was suggested to be aided by an effector triad of the PPi sensor R768 on helix H, the linker D617, and the effector V714-O in an induced-fit mechanism aided by D613 for positioning of the substrate (Fig. 6a) (Baer et al. 2014; Schrepfer et al. 2016). The PPi sensor R768 forms hydrogen bonds with the oxygen atoms of GGPP. It appears that the linker D617 has hydrogen bond interaction with the sensor R768 while D613 has interaction with C2 of GGPP. The ionization of GGPP leads to the formation of the geranylgeranyl cation and the release of PPi in the active site. Secondly, the acyclic geranylgeranyl cation undergoes ring closure to form the monocyclic cembren-15-yl cation. It is revealed that W753, Y835 and Y841 are stabilizing the geranylgeranyl cation through π-π interactions (Fig. 6b) while V714, L716 and W753 are showing hydrophobic interactions with C1 and C2 suggesting their involvement in C1-C2 ring closure (Fig. 6c). Meanwhile, as previously published, the ionized PPi is retained in the active site forming an R-PPi motif (Schrepfer et al. 2016; Freud et al. 2017). In our model, we observe the possibility of an R578-PPi-R754 motif (Fig. 6d) as a candidate for the active site base that can be involved in deprotonation steps along with water-assisted proton transfer to PPi. The cembren-15-yl cation is associated by π-π interactions with W753, F834, Y835 and Y841 and is rapidly converted to the bicyclic verticillen-12-yl cation which is showing π-π interactions with W753 only (Fig. 6e). This reduced π-interactions might be one of reasons for the promiscuous production of verticillene-type compounds. Also, cation–π interaction is observed between C3–C4 double bond and the positively charged C12 which in turn show ionic interaction with D613 (Fig. 6f). These interactions probably contribute to the formation of the next carbocation in the cascade, verticillen-4-yl cation (Fig. 1). We limited our focus on the mechanistic insight into the first half of the TXS reaction cascade.

**Fig. 7:**
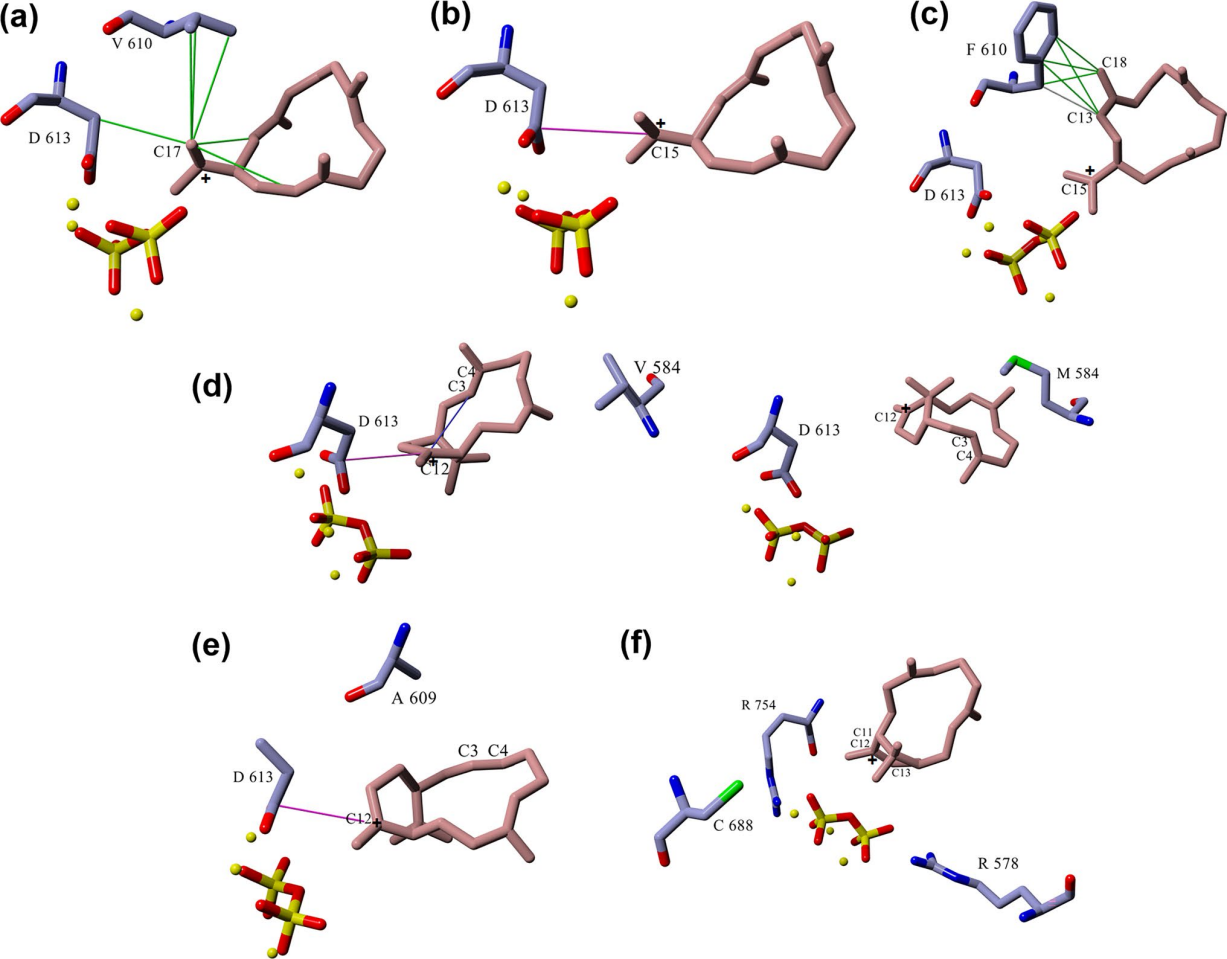
Docked cembren-15-yl cation in wild-type TXS (**a**, **b**) and V610F mutant (**c**), and docked verticillen-12-yl cation in wild type and V584M (**d**), Q609A (**e**), and Y688C (**f**)

A closer look into selected mutants that produce either cembrene A or V as the major product was warranted. V610F produced cembrene A as the major product (68%) with minor production of V (5.3%) and V1 (8.54%) while taxa-4(5)-diene and taxa-4(20)-diene are no longer produced. To better understand the deprotonation event of the cembren-15-yl cation leading to the formation of cembrene A, MD simulation comparison of the docked cembren-15-yl cation in both wild-type TXS and V610F mutant was performed. In wild-type TXS, the V610 residue is showing hydrophobic interaction with C17 of the cembren-15-yl cation (Fig. 7a). In addition, D613 has ionic interaction with the positively charged C15 (Fig. 7b), probably hindering deprotonation toward formation of cembrene A and allowing transition to the next carbocation in the reaction cascade. However, the MD simulation of the V610F mutant with cembren-15-yl cation showed that the F610 residue no longer interacts with C17 of the cation freeing its methyl-hydrogens taking away the obstruction to form cembrene A. F610 instead interacts with C13 and C18 (Fig. 7c) and along with the fact that the phenylalanine residue is aromatic, it contributes to the already existing stabilizing π–π interactions of the cembren-15-yl cation. Also, D613 and C15 are no longer interacting, combined with the now free C17 methyl-hydrogens that are shifted by ≈ 1 Å in closer proximity to the R578-PPi-R754 motif (Fig. 7c) which allows easier deprotonation of the cation compared to the wild type. In addition, sequence alignment with cembrene A synthase revealed an aromatic tyrosine residue in this position (Fig. S2). Hence, V610F showed much higher production of cembrene A relative to the wild type where it was undetectable. This premature interruption of the reaction cascade explains why no taxadiene products are formed.

As for mutants with verticillia-3(4),7(8),12(13)-triene (V) as the major product, V584M, Q609A, and Y688C showed marked decrease in the production of taxa-4(5)-diene with increased proportion of V; 89%, 77% and 69%, respectively. MD simulation comparison of the docked verticillen-12-yl cation in both wild-type TXS, V584M, Q609A and Y688C mutants was performed. V584 is in close proximity of the verticillen-12-yl cation showing several hydrophobic interactions. In V584M, the cation–π interaction between C3–C4 double bond and the positively charged C12 which in turn shows ionic interaction with D613, observed in wild type, is no longer present (Fig. 7d). The distance from R578-PPi-R754 motif did not change significantly; however, restrictions present on the cation were reduced allowing deprotonation at C13, whether by the motif or water-assisted deprotonation, and production of V in high amounts. As for Q609, hydrophobic interactions with C20, in addition to the previously discussed interactions, hold the verticillen-12-yl cation in position and prevent it from flipping. Q609A lacks interaction with C20, in addition to the disappearance of the cation–π interaction between C3–C4 double bond and the positively charged C12. The less bulky alanine residue allows more free movement of the cation where at certain points of the MD simulation, the hydrogens of C13 shift closer to the R578-PPi-R754 motif facilitating deprotonation and formation of V (Fig. 7e). Finally, Y688 residue does not interact directly with the cation but with neighboring amino acid residues. All Y688 mutants showed high degrees of promiscuity indicating that its mutation affects the environment surrounding the cation. Among them, Y688C produced the highest amount of V as the major product. Similar to the previously discussed mutants, the cation–π interaction between C3–C4 double bond and the positively charged C12 which in turn shows ionic interaction with D613 is not present in Y688C. Also, the C13 hydrogens shifted by ≈ 2 Å closer to the R578-PPi-R754 motif assisting deprotonation and formation of V (Fig. 7f).

Creating higher order mutants to improve catalytic activity is usually considered. Double mutants to increase the yield of the desired product, taxa-4(20)-diene, cembrene A or verticillia-3(4),7(8),12(13)-triene (V), by improving TXS activity were attempted. For example, a double mutant of Y688L and Y762F was created to attempt to improve production of taxa-4(20)-diene. However, the preliminary results showed no increase in catalytic activity compared to the single mutants. Future research can include trials of double and triple mutants incorporating the discussed residues. The challenge would be discovering a successful mutant combination to achieve the desired effect especially that improving TXS activity is not easily attained.

## Conclusion

Mechanistic insight into the catalysis cascade of TXS was achieved. TXS reaction cascade starts with ionization of GGPP through induced-fit mechanism via an effector triad of the PPi sensor R768, the linker D617, and the effector V714-O. Following the initial ionization of GGPP, the geranylgeranyl cation and PPi are both separated and correlated to residues in the active site. The geranylgeranyl cation and cembren-15-yl cation are engaged in π–π interactions with several aromatic residues in the active site while π interactions are reduced with the verticillen-12-yl cation. Single amino acid substitution can lead to premature interruption of the reaction cascade reducing or abolishing the production of taxanes in favor of cembranoid-type and/or verticillene-type side products which is in direct relation to the promiscuity of TXS. The mutability landscape has allowed us to unravel that residues V584, Q609, V610, and Y688 are most important for selectivity, and mutants in these positions play a crucial role in premature termination of the reaction cascade.

From a perspective of high yield (in microbial cell factories), mutants with increased production of taxa-4(20)-diene may be beneficial for improved production of Taxol. However, increased percentage of taxa-4(20)-diene relative to taxa-4(5)-diene was accompanied by reduced catalytic activity of several of these mutants compared to wild-type TXS. Hence, future engineering to improve their catalytic activity should be considered to boost the amount of taxa-4(20)-diene produced. Mutants with good catalytic activity and higher production of taxa-4(20)-diene should be carefully examined for increased production of other minor cembranoid-type and verticillene-type compounds as these compounds do not contribute to the production of Taxol. Thus, we suggest that the best candidates would be mutants displaying higher production of taxa-4(20)-diene while sustaining high total production with taxa-4(5)-diene or mutants that produce taxa-4(20)-diene as the major product in a high proportion.

### Supplementary Information

Below is the link to the electronic supplementary material.

Supplementary file1 (PDF 1503 KB)

## Data Availability

All the data supporting the findings of this study are included in this article/Supplementary information. Inquiries about the data generated and analyzed can be directed to the corresponding author.

## Abbreviations

GGPP: Geranylgeranyl pyrophosphate
MD: Molecular dynamics
PPi: Pyrophosphate
Taxa-4(5)-diene: Taxa-4(5),11(12)-diene
Taxa-4(20)-diene: Taxa-4(20),11(12)-diene
TXS: Taxadiene synthase
V: Verticillia-3(4),7(8),12(13)-triene
V1: Verticillia-4(20),7(8),11(12)-triene
V2: Verticillia-3(4),7(8),11(12)-triene
