# Supplementary material for: Insights into taxadiene synthase catalysis and promiscuity facilitated by mutability landscape and molecular dynamics
Source: Planta. 2024 Mar 9;259(4):87. doi: 10.1007/s00425-024-04363-9 (PMC10924717; doi:10.1007/s00425-024-04363-9)
Supplement: Supplementary file 1 — Supplementary file1 (PDF 1503 KB) [file 425_2024_4363_MOESM1_ESM.pdf]

Insights into taxadiene synthase catalysis and promiscuity facilitated by mutability landscape and molecular dynamics

Planta

Siqi He[1,†], Ingy I. Abdallah[1,2,†], Ronald van Merkerk[1], Wim J. Quax[1,*]

[1]Department of Chemical and Pharmaceutical Biology, Groningen Research Institute of Pharmacy, University of Groningen, Antonius Deusinglaan 1, 9713 AV Groningen, The Netherlands

[2]Department of Pharmacognosy, Faculty of Pharmacy, Alexandria University, Egypt

[†]Siqi He and Ingy I. Abdallah should be considered joint first author.

***Corresponding author:** Prof. Dr. Wim J. Quax

Address: Antonius Deusinglaan 1, Building 3215, room 917, 9713AV, Groningen, The Netherlands

Tel: +31 (0) 50 363 2558, (0) 50 363 8174

Fax: +31 (0) 50 363 3000

E-mail: w.j.quax@rug.nl

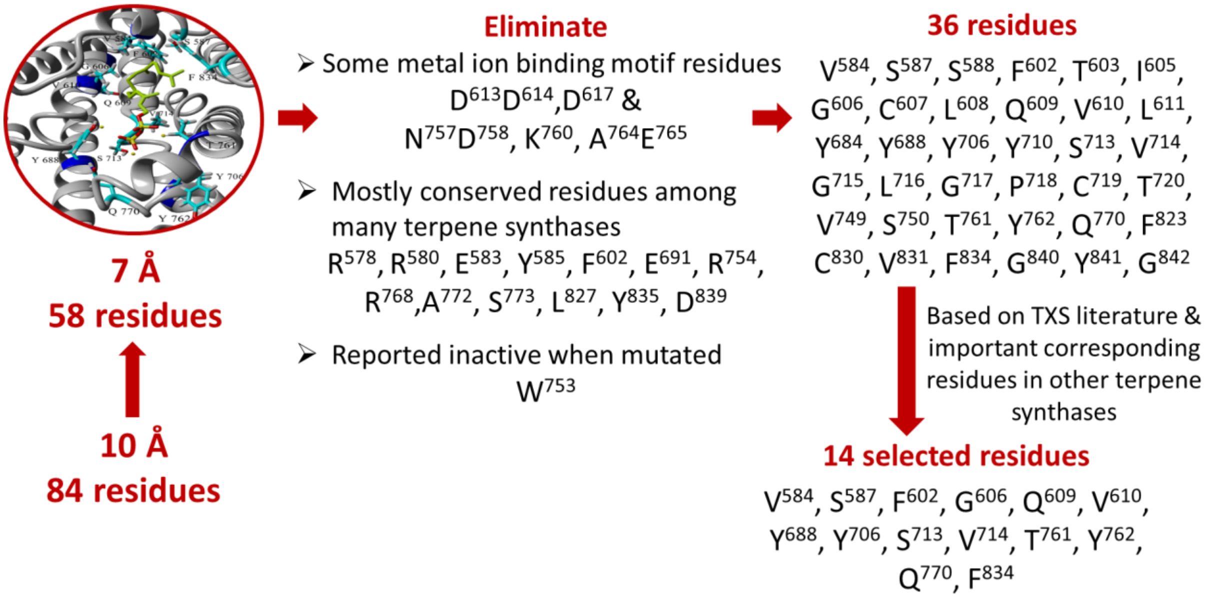

Fig. S1 Selection of candidate residues for mutation

Diterpene synthase class I	Taxadiene synthase	V584	S587	F602	G606	Q609	V610	Y688	Y706	S713	V714	T761	Y762	Q770	F834
Diterpene synthase class I	Cembrene A synthase	A	Y	N	L		Y	L	M	D	A	S	Y	D	I
Diterpene synthase class II	Ent-copalyl diphosphate synthase	C	L	W	S	V	K	G	W	G	E				F
Diterpene synthase class I & II	Abietadiene synthase	I	S	Y	S	T	V	Y	Y	S	I	T	Y	E	F
Sesquiterpene synthase	E-α-bisabolene synthase	F	L	F	A	Q	T	Y	Y	S	I	T	Y	E	I
Sesquiterpene synthase	5-*epi*-aristolochene synthase	C	W	L	I	I	S	Y	Y	T	T	T	Y	Q	T
Sesquiterpene synthase	Amorphadiene synthase	C	W	F	V	I	T	L	H	T	G	T	H	H	Q
Sesquiterpene synthase	Germacrene synthase	I	W	A	T	L	V	Y	Y	T	S	T	Y	Q	V
Sesquiterpene synthase	α-humulene synthase	C	W	T	I	M	S	Y	H	T	A	S	H	H	I
Sesquiterpene synthase	Epi-cedrol synthase	C	W	L	F	Q	T	Y	H	S	I	S	H	H	L
Sesquiterpene synthase	Aristolochene synthase	C	W	L	I	I	S	Y	Y	T	T	T	Y	Q	T
Sesquiterpene synthase	γ-cadinene synthase	G	W	L	I	A	S	Y	F	T	C	E	H		L
Monoterpene synthase	Limonene synthase	C	W	M	N	I	T	Y	Y	S	I	T	S	D	M
Monoterpene synthase	Bornyl diphosphate synthase	S	W	A	I	A	T	Y	Y	S	V			D	I

Fig. S2 Multiple sequence alignment of the mutability landscape of TXS with three diterpene synthases, eight sesquiterpene synthases and two monoterpene synthases. Residues that are identical, strongly similar, weakly similar and non-matching compared to TXS are given the colors dark blue, blue, cyan and white, respectively.

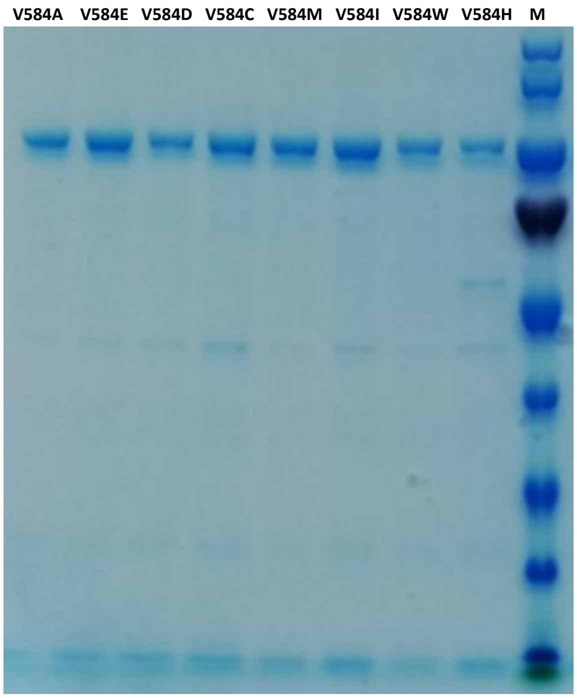

Fig. S3 SDS-gel of some TXS V584 mutants showing purified his-tagged protein (92.6 kDa).

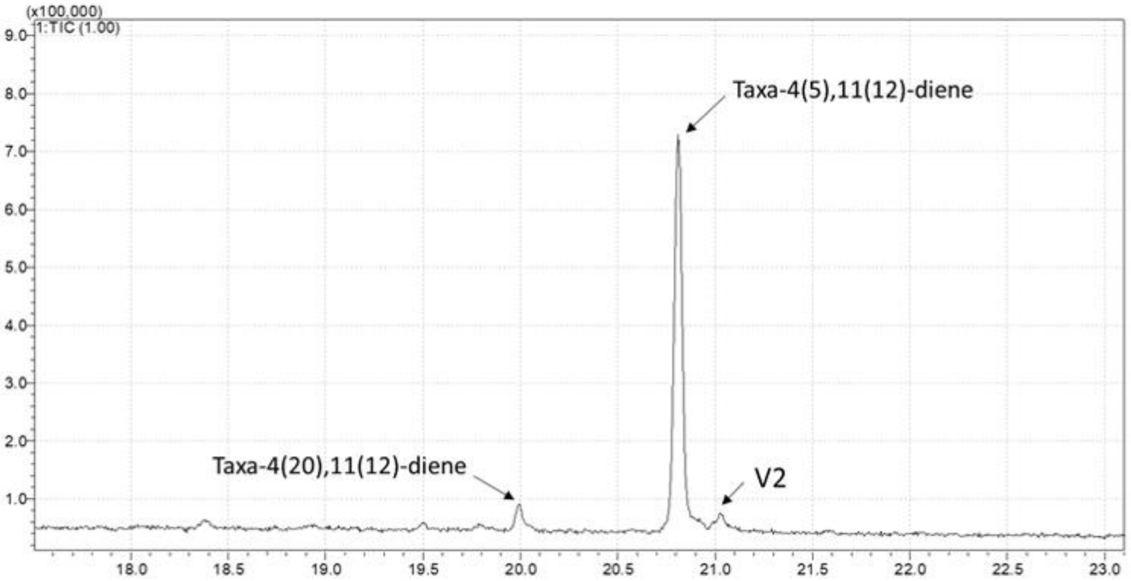

Fig. S4 GC chromatogram of wild type TXS.

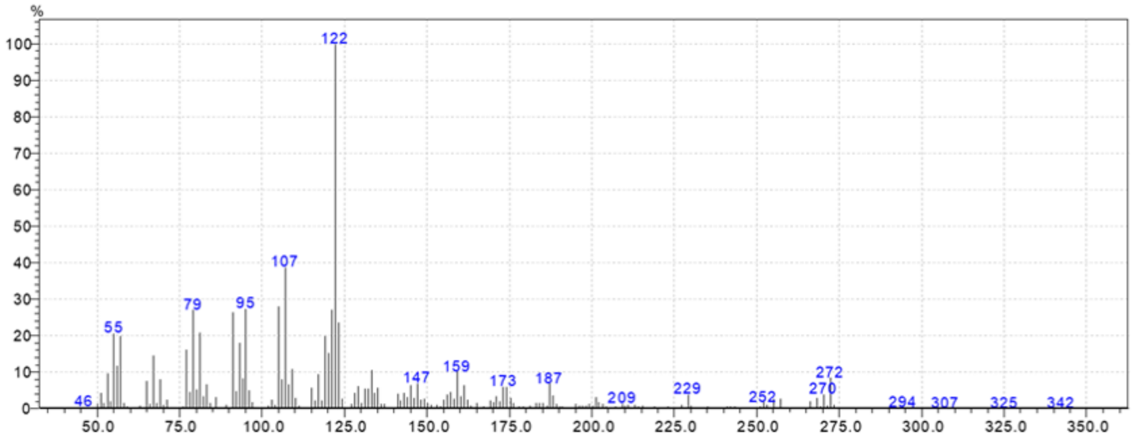

Fig. S5 Mass spectrum of taxa-4(5),11(12)-diene.

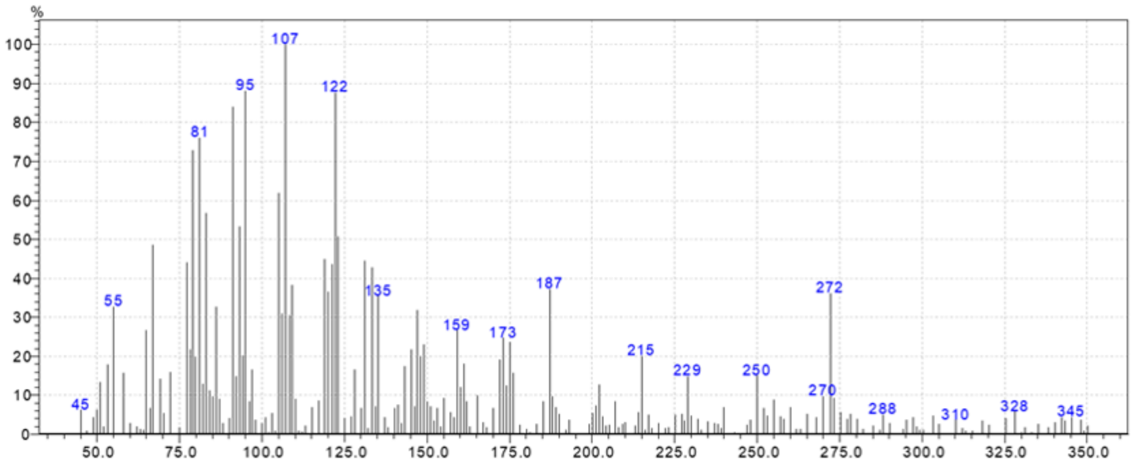

Fig. S6 Mass spectrum of taxa-4(20),11(12)-diene.

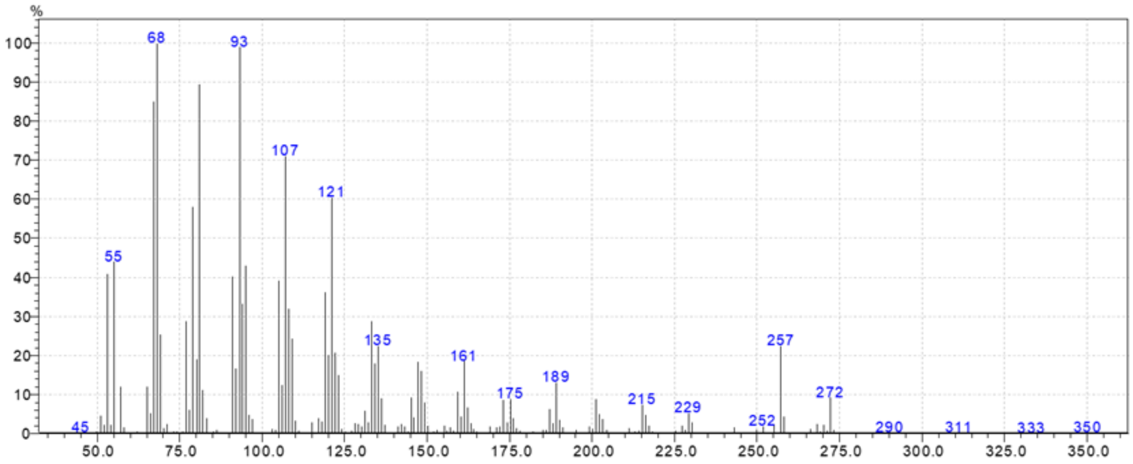

Fig. S7 Mass spectrum of cembrene A.

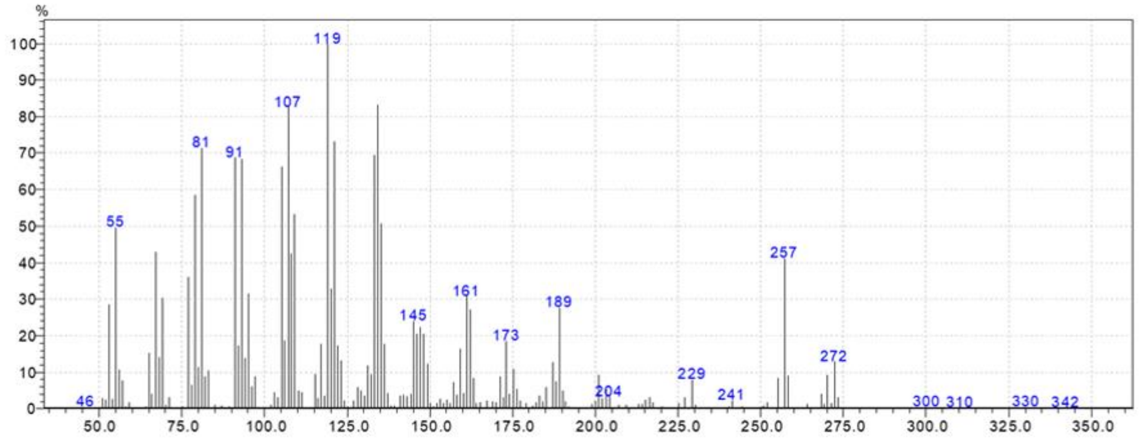

Fig. S8 Mass spectrum of verticillia-3(4),7(8),12(13)-triene (V).

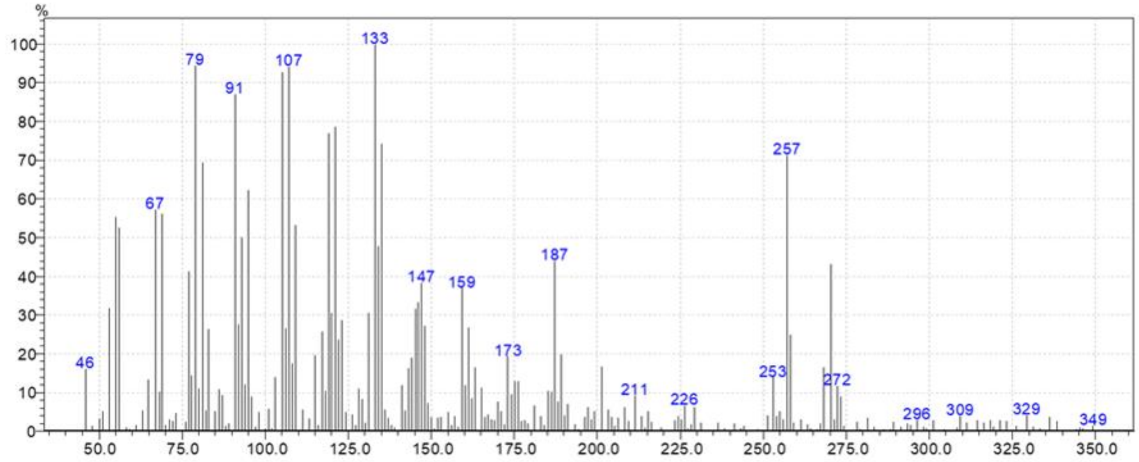

Fig. S9 Mass spectrum of verticillia-4(20),7(8),11(12)-triene (V1).

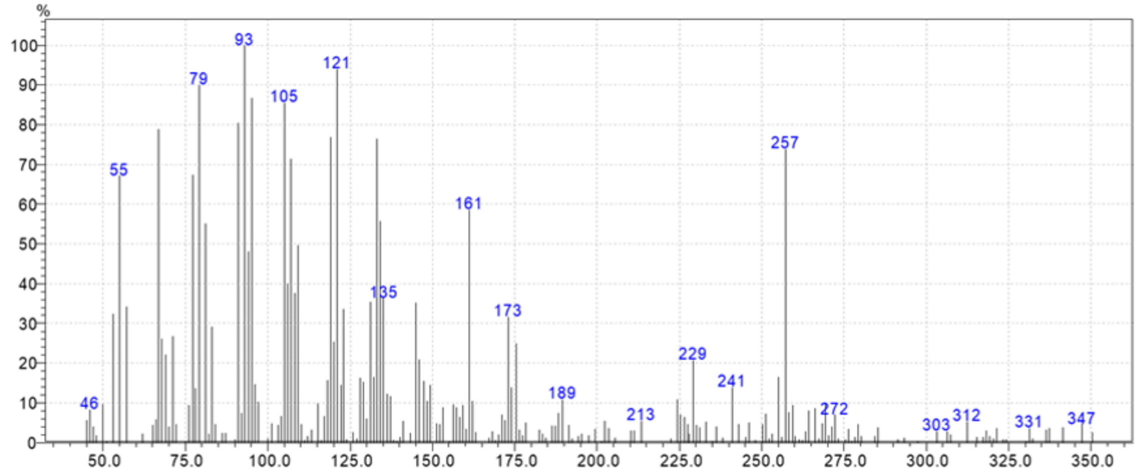

Fig. S10 Mass spectrum of verticillia-3(4),7(8),11(12)-triene (V2).

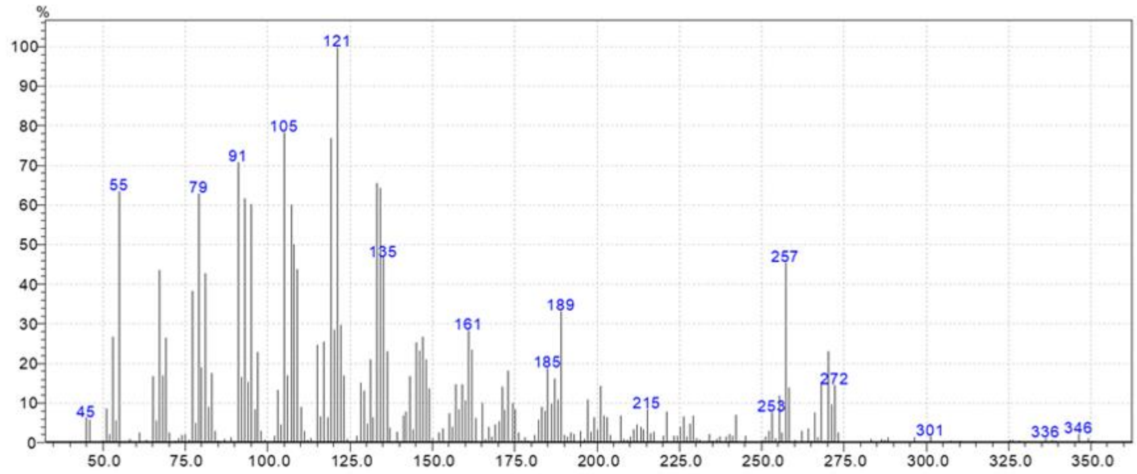

Fig. S11 Mass spectrum of V2 isomer.

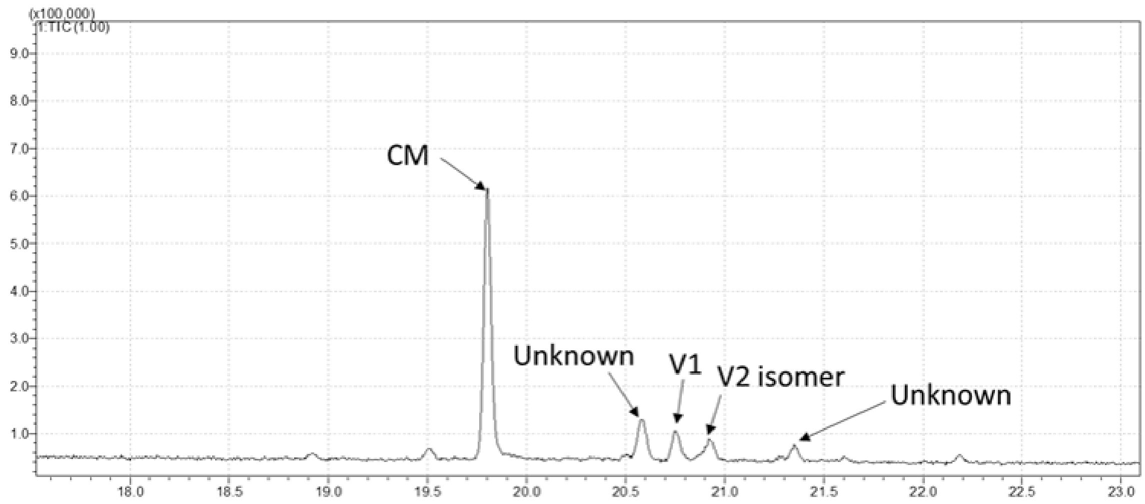

Fig. S12 GC chromatogram of V610F.

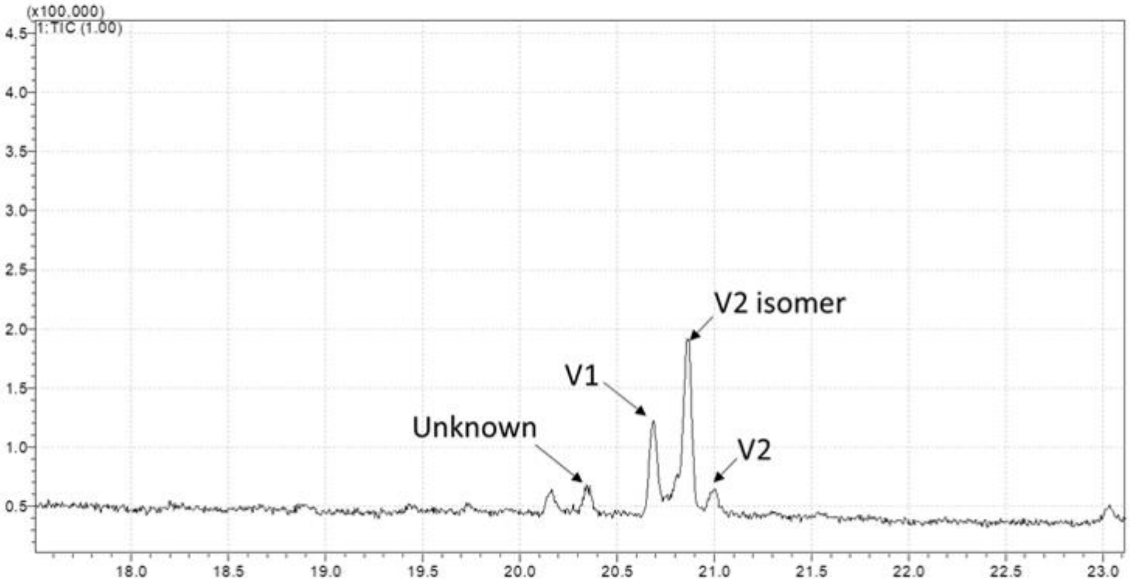

Fig. S13 GC chromatogram of V610M.

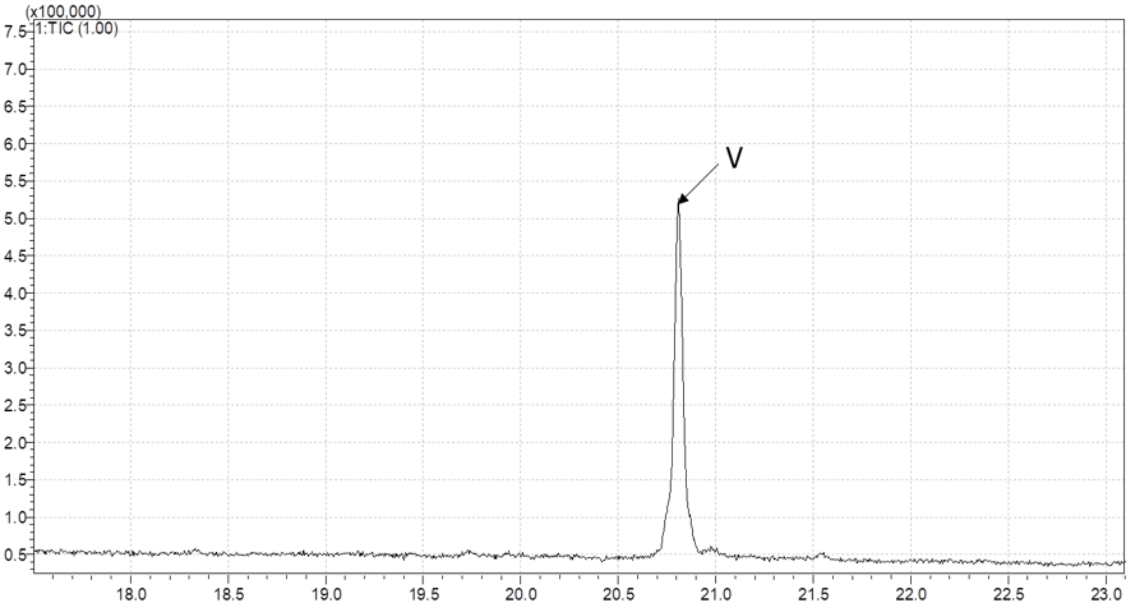

Fig. S14 GC chromatogram of V584M.

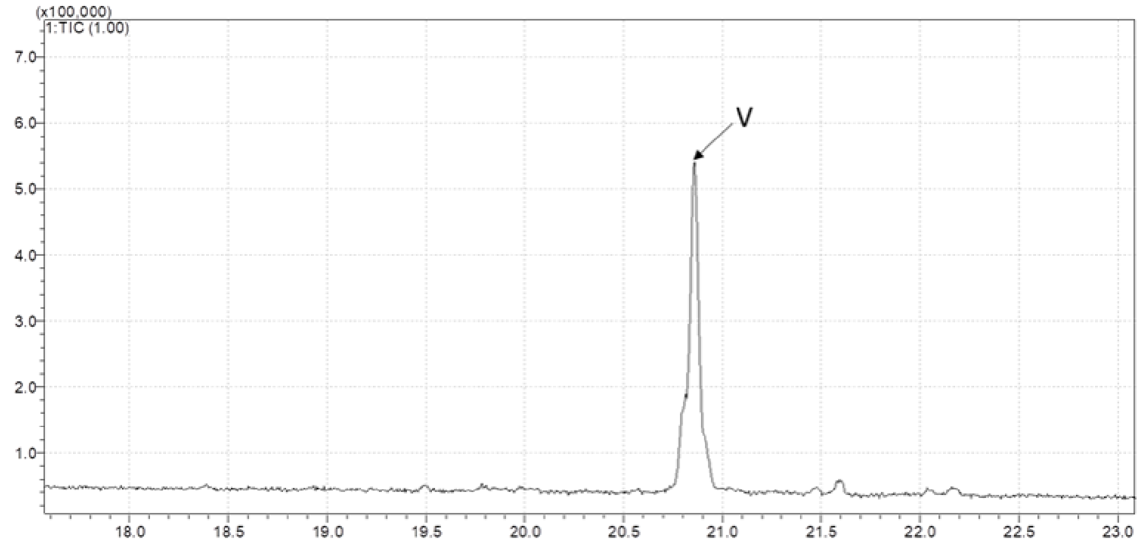

Fig. S15 GC chromatogram of Q609A.

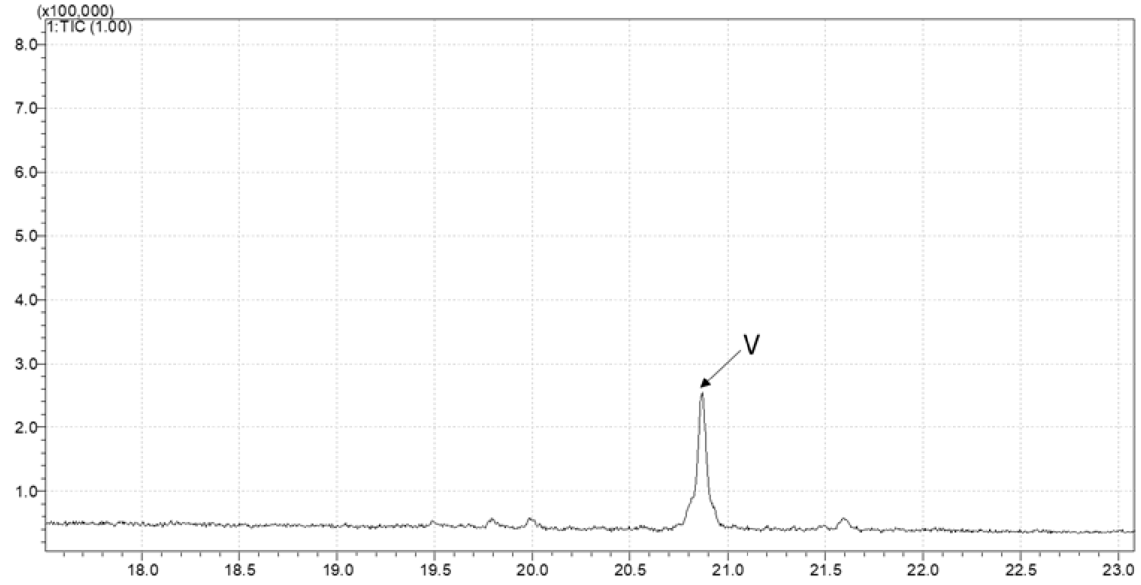

Fig. S16 GC chromatogram of Y688C.
